# Preoperative versus postoperative ultrasound-guided rectus sheath block for acute postoperative pain relief after laparoscopy: A retrospective cohort study

**DOI:** 10.1097/MD.0000000000037597

**Published:** 2024-03-29

**Authors:** Mayuko Nakazawa, Toko Fukushima, Kazuhiro Shoji, Ryo Momosaki, Yasushi Mio

**Affiliations:** a Department of Anesthesiology, Tokyo Jikei University Katsushika Medical Center, Tokyo, Japan; b Department of Anesthesiology, The Jikei University School of Medicine, Tokyo, Japan; c Department of Rehabilitation Medicine, Mie University Graduate School of Medicine, Mie, Japan.

**Keywords:** Postoperative pain, preemptive analgesia, rectus sheath block, ropivacaine

## Abstract

Although rectus sheath block (RSB) is routinely used in laparoscopic surgeries to reduce mid-abdominal pain, whether RSB should be performed before or after surgery remains unclear. Herein, the optimal timing for RSB in patients undergoing laparoscopic surgery was investigated. This retrospective cohort study analyzed the data of patients who underwent RSB during laparoscopic procedures at our hospital between January 2013 and December 2018. The primary outcome was the time to rescue analgesia within 24 hours postanesthesia. The patients were divided into preoperative (pre-) and postoperative (post-) RSB groups. A multivariable Cox proportional hazards regression model was used to analyze the time to rescue analgesia in the unmatched and propensity score (PS)-matched patient populations. In total, 609/14,284 patients were included (pre-RSB group, 227 patients; post-RSB group, 382 patients). After PS matching, 97 patients were assigned to both groups. Although the time from extubation to the first analgesic request was not significantly different between the 2 groups (322 vs 294 minutes, *P* = .57), the patients in the pre-RSB group showed a lower risk of postoperative first analgesic administration after PS matching (adjusted hazard ratio, 0.71; 95% confidence interval, 0.53–0.95; *P* = .023). Among patients undergoing laparoscopic surgery, those in the pre-RSB group tended to have a longer time to the first analgesic request and had a lower risk of analgesic administration within the first 24 hours than those in the post-RSB group. Thus, performing RSB preoperatively may be preferable.

## 1. Introduction

Rectus sheath block (RSB) is an anterior abdominal wall block that reduces postoperative pain associated with midline incisions.^[1]^ We place the RSB between the rectus muscle and its posterior sheath. The injection blocks the anterior cutaneous branches of the lower thoracic spinal nerves (T7–T12), which are responsible for sensory function in the mid-abdominal cutaneous area and the rectus abdominis muscle sensation.^[2]^ The use of RSB in laparoscopic procedures has increased recently.^[3]^ Multiport laparoscopic surgery usually requires 3–5 umbilical or periumbilical incisions. Insertion of a large-bore trocar into the umbilicus requires closure of the peritoneum and fascia; therefore, the umbilical incision site is more painful for the patient, compared to skin sutures alone.^[1]^ RSB is performed for umbilical and periumbilical sites and is effective in reducing umbilical incisional pain during and after surgery.^[4,5]^ Moreover, RSB can be used with abdominal ultrasound because it is associated with fewer complications.^[4]^

At our institution, we perform ultrasound-guided RSB before or after surgery. RSB before surgery is expected to reduce pain during and after the surgery, based on the principle of “preemptive analgesia.”^[6–9]^ In contrast, RSB after surgery is expected to prolong the duration of the effect of the local anesthetic. To the best of our knowledge, few studies have examined the timing of RSB implementation. Therefore, in the present study, we hypothesized that preoperative RSB (pre-RSB) would provide less postoperative analgesia than postoperative RSB (post-RSB) and attempted to verify the appropriate timing of RSB implementation.

## 2. Material and methods

### 2.1. Patient selection

This retrospective cohort study was conducted after obtaining ethical approval from The Jikei University School of Medicine Institutional Review Board (30-287[9308]). This clinical study did not analyze any secondary data. The Institutional Review Board waived the requirement for obtaining informed consent because the procedures did not require any additional intervention and all patient data were anonymous. This study was conducted in accordance with the Strengthening the Reporting of Observational Studies in Epidemiology guidelines^[10]^ and the Declaration of Helsinki at the Department of Anesthesiology of the Tokyo Jikei University Katsushika Medical Center using the opt-out method on the hospital website. Before accessing the data, we posted a document, which was approved by the ethics committee, on the website to provide the participants an opportunity to reject the use of their personal information.

We included adult (≥18 years) patients with surgical, urological, and gynecological issues who underwent laparoscopic surgery at our institution between January 1, 2013, and December 31, 2018. We excluded patients who had received epidural or spinal anesthesia, intravenous patient-controlled analgesia (iv-PCA), postoperative mechanical ventilation, or both pre- and postoperative RSB, as well as those who had undergone conversion to laparotomy. Patient characteristics and intraoperative data were retrieved from the operating room database (Philips Electronics Japan, Tokyo, Japan), intensive care unit database and patient information system (Philips Electronics Japan), and general ward database and patient information system (HOPE EGMAIN-GX; Fujitsu, Kanagawa, Japan).

The primary outcome was the first time to rescue analgesia within 24 hours after completion of anesthesia (time of extubation). Analgesics were administered at the request of the patient in the postanesthesia care unit or the ward and recorded in the electronic medical records; the type of analgesic was determined by the anesthesiologist or surgeon. Intravenous analgesics, including fentanyl, flurbiprofen, acetaminophen, buprenorphine, pentazocine, and tramadol, were evaluated in this study. Oral analgesics were excluded because the records did not include the correct time in which the patients received them. The secondary outcome was the time from surgery completion to discharge (days).

### 2.2. Sample size

We estimated the sample size based on the expected incidence of postoperative usage of analgesic agents, as suggested in previous studies.^[4]^ Assuming that the baseline incidence of the usage of analgesic agents after anesthesia was 75% in the control group and considering a 20% reduction in the incidence in the pre-RSB group to be clinically important, we estimated that 99 patients would be required to demonstrate this difference (two-sided alpha level of 0.05, power of 0.8). We expected approximately 900 eligible patients to visit the medical center during a 6-year period, which allowed us to include up to 12 variables in the multivariate logistic regression analysis.

### 2.3. Adjustment for differences between the groups

The patients were divided into 2 groups (pre- and post-RSB). In our institution, the need for perioperative RSB and the choice between pre- and postoperative RSB were decided by the anesthesiologist and/or their preceptor. The RSBs were performed by trained or trainee anesthesiologists. The RSB performed in this study was almost always bilateral. We hypothesized that the characteristics of the 2 groups would be different, and 1:1 propensity score (PS) matching was used as the primary tool to adjust for differences (caliper, 0.2). A multivariate logistic regression model was constructed to calculate the PS for each participant based on whether RSB was performed before or after surgery.^[11]^ Variables considered to affect the indication for RSB were as follows: age (≥65 years or not); sex; body mass index (BMI) (>30 kg/m^2^ or not); department (general surgery, urology, gynecology); a history of dual antiplatelet therapy (DAPT), defined as the use of aspirin or cilostazol combined with a P2Y12 inhibitor; a history of using oral anticoagulant medications, defined as warfarin or direct oral anticoagulants; presence or absence of end-stage renal disease; predictive operative time (<120, 120–240, and ≥240 minutes); type of anesthesia (sevoflurane, desflurane, total intravenous anesthesia [TIVA], others); and year (2013, 2014, 2015, 2016, 2017, and 2018). We used the C-statistic to evaluate the goodness of fit.^[11]^ Covariate balance before and after PS matching was compared using standardized differences,^[12]^ and standardized mean differences within 10% for all variables were indicative of successful balance.

### 2.4. Statistical analyses

Missing data for categorical variables were assigned to a separate group (categorized as “not applicable”) while missing data for numerical variables were excluded from the analysis. Baseline characteristics for the full cohort are summarized as numbers and percentages for categorical variables, and mean values and standard deviations for continuous variables. Variables before and after PS matching were analyzed to determine between-group differences using the *t* test for continuous variables, the chi-squared test and 2-way analysis of variance for categorical variables, and the log-rank test for Kaplan–Meier curves. Statistical significance was defined as a 2-tailed *P* value < .05.

We conducted multiple Cox regression analyses and multiple logistic regression analyses to identify the association between RSB categories and the use of analgesics after anesthesia for 24 hours. The hazard ratios and odds ratios were calculated after adjustments for the following predictor variables: age (≥65 years or not); sex; BMI (>30 kg/m^2^ or not); department (general surgery, urology, gynecology); history of DAPT; history of using oral anticoagulant medications; emergency; operative time (<120, 120–240, and ≥ 240 minutes); type of anesthesia (sevoflurane, desflurane, TIVA, others); year (2013, 2014, 2015, 2016, 2017, and 2018); and history of using fentanyl, remifentanil, flurbiprofen, acetaminophen, and tramadol during surgery. We performed PS-adjusted Cox regression analysis and logistic regression analysis and obtained Kaplan–Meier curves to adjust for differences in patient characteristics. All statistical analyses were performed using R software (version 4.1.2; R Foundation for Statistical Computing, Vienna, Austria).

## 3. Results

**Figure 1.**
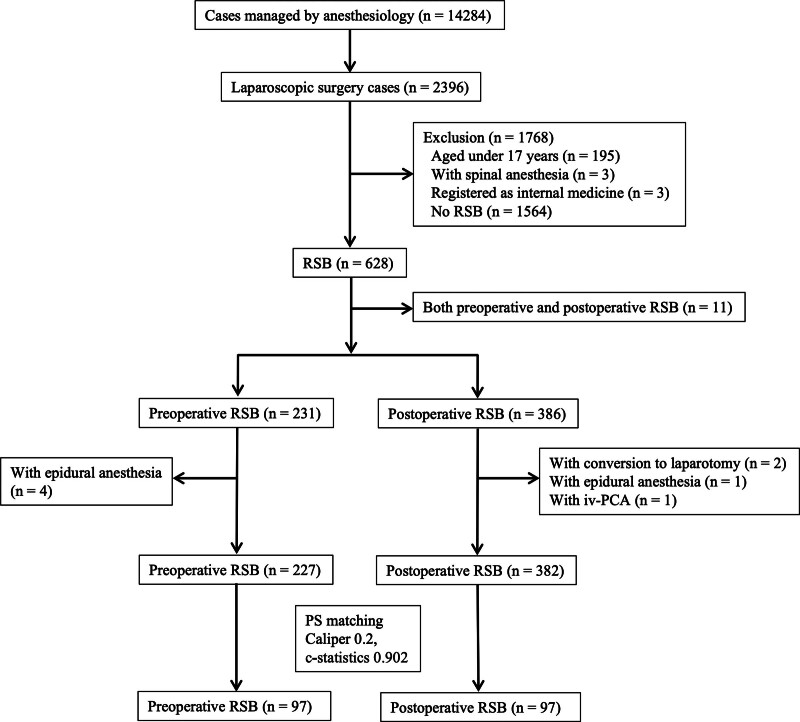
Flowchart of the study population. Among the 2396 patients who underwent laparoscopic surgery between January 2013 and December 2018, 609 were divided into 2 groups (pre-RSB [*n* = 227] and post-RSB [*n* = 382]). After 1:1 PS matching (caliper = 0.2, C-statistic = 0.902), patients were assigned to the pre-RSB (*n* = 97) and post-RSB (*n* = 97) groups. iv-PCA = intravenous patient-controlled analgesia, PS = propensity score, pre-RSB = preoperative rectus sheath block, post-RSB = postoperative rectus sheath block.

From January 1, 2013, to December 31, 2018, 2396 of 14,284 patients underwent laparoscopic surgery, and 628 patients met the inclusion criteria. After excluding 11 patients who had undergone both pre- and post-RSB, 231 and 386 patients were allocated to the pre-RSB and post-RSB groups, respectively. Eight patients were excluded because they received epidural anesthesia or iv-PCA or had undergone conversion to laparotomy. Finally, 227 and 382 patients were included in the pre- and post-RSB groups, respectively. A total of 51 data points were missing for 3 variables (BMI, predictive operative time, and operative time). Consequently, we performed 1:1 PS matching with a caliper of 0.2 and a C-statistic of 0.902, and patients were assigned to the 2 groups (pre-RSB [*n* = 97] and post-RSB [*n* = 97]) (Fig. 1).

Patient characteristics are presented in Table 1. Before PS matching, the patients in the pre-RSB group were younger, predominantly male, had larger mean BMI, and more frequently underwent emergency surgery than those in the post-RSB group. Bias was also observed in relation to the department; more patients were from the gynecology department and fewer patients were from the surgery and urology departments in the pre- than in the post-RSB group (SMD = 0.26). Particular bias was observed according to year, with more patients in 2018 in the pre- than in the post-RSB group (SMD = 1.57). After PS matching, the distribution of years was well-balanced between the 2 groups (SMD = 0.07). However, age, BMI, and percentage of patients in the departments were not well-balanced between the 2 groups (age, SMD = 0.14; BMI, SMD = 0.17; departments, SMD = 0.16). In both groups, 0.2% ropivacaine was mainly used for local anesthesia. Desflurane-based anesthesia was more frequently performed in the pre- than in the post-RSB group; while the use of acetaminophen was more frequent during surgery in the pre-RSB group; the use of fentanyl, remifentanil, and flurbiprofen was less frequent. These differences were observed both before and after PS matching.

**Table 1:** Patient characteristics.

Factor	Before PS matching			After PS matching		
	Pre-RSB	Post-RSB	SMD	Pre-RSB	Post-RSB	SMD
Number of patients	227	382		97	97	
Age, y	47.4 (15.7)	51.4 (17.4)	0.25	50.8 (16.6)	48.6 (16.6)	0.14
Male, N	51 (22.5)	137 (35.9)	0.30	22 (22.7)	25 (25.8)	0.07
BMI, kg/m^2^	22.6 (3.7)	23.6 (4)	0.26	22.8 (4.3)	23.5 (3.6)	0.17
Emergency, N	63 (27.8)	57 (14.9)	0.32	21 (21.6)	19 (19.6)	0.05
Department, N						
Gynecology	116 (51.1)	142 (37.2)	0.26	43 (44.3)	50 (51.5)	0.16
Surgery	109 (48)	208 (54.5)		52 (53.6)	46 (47.4)	
Urology	2 (0.9)	32 (8.4)		2 (2.1)	1 (1)	
Year, N						
2013	0 (0)	4 (1)	1.57	0 (0)	0 (0)	0.07
2014	0 (0)	4 (1)		0 (0)	0 (0)	
2015	0 (0)	49 (12.8)		0 (0)	0 (0)	
2016	3 (1.3)	83 (21.7)		2 (2.1)	3 (3.1)	
2017	47 (20.7)	167 (43.7)		41 (42.3)	42 (43.3)	
2018	177 (78.0)	75 (19.6)		54 (55.7)	52 (53.6)	
RSB						
Type of local anesthetic, N						
0.2% Ropivacaine	178 (78.4)	293 (76.7)	0.13	73 (75.3)	70 (72.2)	<0.01
0.25% Ropivacaine	10 (4.4)	20 (5.2)		5 (5.2)	6 (6.2)	
0.3% Ropivacaine	39 (17.2)	66 (17.3)		19 (19.6)	21 (21.6)	
Dosage of local anesthetic, mL	38.6 (10)	30.3 (12.3)	0.74	38 (10.8)	34.2 (11.5)	0.34
Type of anesthesia, N			0.156			0.20
Sevoflurane, N	86 (37.9)	161 (42.1)		41 (42.3)	46 (47.4)	
Desflurane, N	101 (44.5)	153 (40.1)		44 (45.4)	37 (38.1)	
TIVA, N	37 (16.3)	67 (17.5)		12 (12.4)	13 (13.4)	
Others, N	3 (1.3)	1 (0.3)		0 (0.0)	1 (1.0)	
Intraoperative analgesic use						
Fentanyl						
Patients, N	115 (50.7)	213 (55.8)	0.10	48 (49.5)	53 (54.6)	0.10
Dosage, μg	132 (155)	160 (187)	0.17	121 (149)	178 (209)	0.32
Remifentanil, N	201 (88.5)	365 (95.5)	0.26	78 (80.4)	94 (96.9)	0.54
Acetaminophen, N	112 (49.3)	152 (39.8)	0.19	49 (50.5)	41 (42.3)	0.17
Flurbiprofen, N	3 (1.3)	20 (5.2)	0.22	2 (2.1)	5 (5.2)	0.17
Duration of surgery, min	135 (72.2)	125 (68.3)	0.14	135 (72.2)	125 (68.3)	0.14
Duration of anesthesia, min	165 (56.3)	204 (81.3)	0.57	168 (59.3)	197 (71.9)	0.44

Data are expressed as means (standard deviation) or number of patients (percentage).

BMI = body mass index, PS = propensity score, RSB = rectus sheath block, SMD = standardized mean difference, TIVA = total intravenous anesthesia.

**Figure 2.**
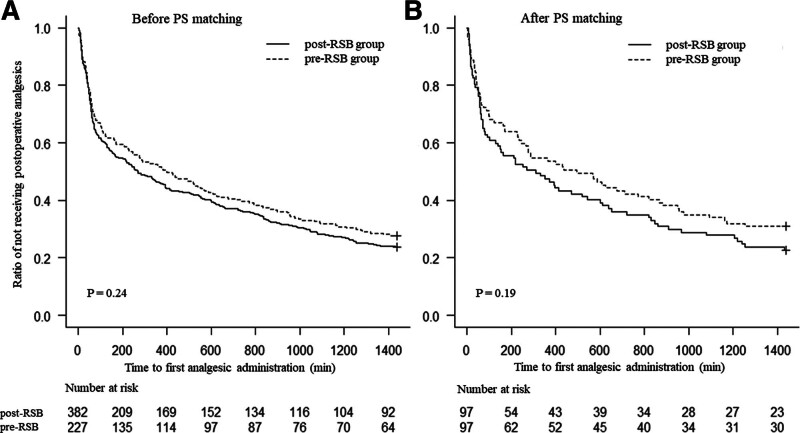
Survival curves for patients who did not receive postoperative analgesics. Before and after PS matching, variables were analyzed for between-group differences using the log-rank test for Kaplan–Meier curves. The dotted and solid lines show the preoperative and postoperative groups, respectively. (A) Patients before and (B) after PS matching. PS = propensity score, RSB = rectus sheath block.

The primary outcome showed no significant differences between the 2 groups before and after PS matching (Table 2). The time from extubation to the first analgesic request tended to be longer in the pre- than in the post-RSB group after PS matching (322 vs 294 minutes, *P* = .57). The findings also showed a trend toward fewer patients in the pre-RSB group not receiving first analgesics within 24 hours (67 vs 74 patients, *P* = .26). Kaplan–Meier curves for the ratio of analgesia-free patients are shown in Figure 2. The time to the first analgesic request was longer in the pre-RSB group, especially since this trend was observed more frequently after PS matching. Cox proportional hazards analysis was performed, which showed that the patients in the pre-RSB group had a lower risk of postoperative first analgesic administration after PS matching (adjusted HR, 0.71; 95% confidence interval, 0.53–0.95, *P* = .023) (Table 3). Analysis of the secondary outcome showed no significant difference in the duration of postoperative hospital stay between the 2 groups (5.9 vs 5.0 days, *P* = .26) (Table 2).

**Table 2:** Postoperative outcomes.

	Before PS matching			After PS matching		
	Pre-RSB	Post-RSB	*P* value	Pre-RSB	Post-RSB	*P* value
Primary outcome						
Number of first time to rescue analgesias within 24 h	164 (72.2)	291 (76.2)	.29	67 (69.1)	75 (77.3)	.26
Time until the first time to rescue analgesia^a^, min	318 (378)	302 (375)	.66	322 (359)	294 (404)	.67
Secondary outcome						
Duration of postoperative hospital stay, days	5.5 (5.0)	6.28 (8.0)	.19	5.9 (5.7)	5.0 (4.9)	.26

PS = propensity score, RSB = rectus sheath block.

a The time was defined as the period from the time of extubation to the time of the first analgesic. Data are expressed as means (standard deviation) or counts (percentage).

**Table 3:** Multivariate Cox proportional hazards analysis for the risk of postoperative analgesic administration.

	Before PS matching			After PS matching		
	HR	95% CI	*P* value	HR	95% CI	*P* value
Pre-RSB	0.82	0.63–1.08	.16	0.71	0.53–0.95	.023

The confounding factors were as follows: age (≥65 years), sex, body mass index (more than 30 or not), department (general surgery, urology, and gynecology), receipt of dual antiplatelet therapy before surgery, anticoagulant medication before surgery, emergency, renal replacement therapy, intraoperative analgesics (fentanyl, remifentanil, acetaminophen, flurbiprofen, and tramadol), duration of anesthesia (<120 min, 120–240 min, and ≥ 240 min), type of anesthesia (sevoflurane, desflurane, total intravenous anesthesia, and others), and year (2013, 2014, 2015, 2016, 2017, and 2018).

CI = confidence interval, HR = hazard ratio, pre-RSB = preoperative rectus sheath block, PS = propensity score.

## 4. Discussion

In the present retrospective cohort study, we performed a multivariate Cox regression analysis of the first postoperative time to rescue analgesia in patients with surgical, gynecological, and urologic issues who underwent laparoscopic surgery. The first time to rescue analgesia after the end of anesthesia was set as the primary outcome and the time to the first analgesic administration within 24 hours was analyzed using objective data. Although the mean time was not significantly different between the 2 groups, the patients in the pre-RSB group had a lower risk of first postoperative analgesic administration within 24 hours after PS matching. To the best of our knowledge, no previous studies examining the timing of RSB have studied the time to first analgesic administration.

Several studies have suggested that pre-RSB is more effective than post-RSB. Yagi et al^[4]^ published a prospective observational cohort study of patients undergoing abdominal wall (transverse abdominal plane [TAP]) block and RSB in total laparoscopic hysterectomy. They showed that nonsteroidal inflammatory drug usage in women who underwent preoperative TAP and RSB was significantly lower than that in women who received a postoperative block within the first 12 hours, with both groups receiving 40 mL of 0.25% ropivacaine in total (pre- vs post-, 54.4% vs 75.0%; *P* = .007).^[4]^ Jeong et al reported a single-center randomized controlled trial on the use of RSB in laparoscopic cholecystectomy.^[6]^ They showed that total rescue analgesic consumption at 3 points up to 24 hours after surgery was significantly lower in the pre- than in the post-RSB group (1 hour, *P* = .023; 9 hours, *P* = .020; 18 hours, *P* = .002), in which patients received 40 mL of 0.25% levobupivacaine.^[6]^ In a meta-analysis of RSB in the setting of laparoscopic surgery by Hamid et al,^[3]^ which included nine randomized controlled trials and 698 patients, pain control was better in the pre-RSB group than in the post-RSB group in a subgroup analysis (*P* = .001). However, these previous studies, as well as the meta-analysis, evaluated specific procedures. In contrast, the present study included patients from many surgical departments. Furthermore, previous studies have used quantitative pain assessments (i.e., pain scores and number of total analgesics) as primary outcomes and assessed them over a certain period, whereas the present study assessed the primary outcome over time using Kaplan–Meier curves and Cox regression.

RSB was performed approximately 2 hours earlier in the pre-RSB group than in the post-RSB group. Initially, we predicted that physicians who performed pre-RSB would intend to provide intraoperative analgesia rather than postoperative analgesia and that physicians who performed post-RSB would intend to provide first postoperative analgesia later. However, the results of the present study contradicted these forecasts. The Kaplan–Meier curve was roughly parallel and showed that the ratio of analgesia-free patients changed at approximately 2 hours after surgery, suggesting that the intergroup differences in the time to rescue analgesia in the present study were earlier than those in previous studies. In previous studies, the time to rescue analgesia was only compared at fixed points, such as 1, 12, and 24 hours after surgery, and differences in requests were examined only at these points.^[3,4,6]^ However, the curves in the present study provide a clearer picture of the differences in the changes between the 2 groups over time.

Although some reports have suggested that pre-RSB is more effective than post-RSB for postoperative analgesia, we could not find any reports concluding that post-RSB is more effective.^[3,4,6]^ We presume that pre-RSB functioned as preemptive analgesia in the present and previous studies. Preemptive analgesia is a form of antinociceptive treatment that prevents the establishment of altered processing of afferent input, which amplifies postoperative pain.^[8]^ In addition, although there are various definitions of preemptive analgesia, we defined it as analgesia that prevents central sensitization caused by incisional and inflammatory injuries (covering the period of surgery and the initial postoperative period).^[8]^ Animal studies have shown that preemptive analgesia can prevent the development of central hypersensitivity after nociceptive demands and reduce postoperative pain.^[13,14]^ Previous studies on patients undergoing laparoscopic surgery have also suggested that pre-RSB provides preemptive analgesia.^[3,4,6]^ Although the concept of preemptive analgesia remains controversial,^[7,9]^ it is supported by the results of the present study.

Notably, 0.2% ropivacaine was mainly used in this study. Ropivacaine (0.25% or higher) was usually used in previous studies that verified ultrasound-guided RSB with ropivacaine in laparoscopic surgery^.[1,15,16]^ The pharmacokinetics of local anesthetics in RSB are not well understood, and the optimal analgesic concentration is also a topic of debate. Abdul Jalil et al^[17]^ reported that 0.2% ropivacaine was as effective as 0.5% ropivacaine in postoperative analgesia with a TAP block in patients undergoing appendicectomy. However, 0.2% ropivacaine is generally accepted as the optimal concentration required for postoperative analgesia in regional anesthesia techniques, except for the abdominal wall.^[18]^ Therefore, we suppose that low-concentration local anesthesia (0.2% ropivacaine) is sufficiently effective for nociception of the umbilical region in laparoscopic surgery.

The patient characteristics in the present study were not well-balanced after PS matching, which may be attributable to the presence of an inappropriate covariate between the 2 groups before PS matching. Covariates that are strongly related to the intervention and unrelated to the outcome are suggested to increase the C-statistic (area under the receiver operating characteristic curve for the probability of intervention) and increase the nonoverlapping distributions of PSs between the treated and untreated groups, resulting in a reduction in the number of matched pairs for analysis.^[19]^ The C-statistic in the present study was high (>0.9), suggesting that such covariates had been included in the multivariate logistic regression model to calculate the PS. The difference in the number of years was too large between the 2 groups before PS matching (absolute standardized difference [ASD], 1.57); thus, strongly affecting the indication for RSB. We presumed that using the year as a covariate was the reason behind the large C-statistic; however, we could not exclude it from the covariates. Apart from the indication for RSB, the year of surgery also affected postoperative time to rescue analgesia because we adopted acetaminophen for injection in 2017, and more than a year passed until it became the usually prescribed drug at our institution. As a result, the number of PS distributions overlapped, the number of matched pairs decreased (Supplementary Figure 1, http://links.lww.com/MD/L973), and the balance of characteristics after PS matching was not optimal. In addition, some characteristics during surgery were not well-balanced. The intraoperative fentanyl and remifentanil dosages were lower in the pre- than in the post-RSB group (fentanyl, 49.5% vs 54.6%, ASD = 0.10; remifentanil, 80.4% vs 96.9%, ASD = 0.54), suggesting that the preoperative RSB may have reduced the need for analgesics during the surgery. In contrast, acetaminophen use was higher in the pre-RSB group (50.5% vs 43.3%, ASD = 0.17). In our institution, we tend to administer acetaminophen immediately before the end of surgery for postoperative analgesia. Before this study, we considered that postoperative administration of analgesics would be more effective for postoperative analgesia because of the longer duration of the effect of local anesthetics. Therefore, a higher percentage of patients in the pre-RSB group may have received acetaminophen as an adjunct to postoperative analgesia in advance. The duration of anesthesia was longer in the post-RSB group (168 vs 197 minutes, ASD = 0.44). We assumed that it was more difficult to perform RSB owing to pneumoperitoneum and the presence of dressing material in the post-RSB group. Although the characteristics of both groups were not well-balanced, we analyzed them including unbalanced covariates with multivariable Cox regression after PS matching to eliminate the effects of confounders. We demonstrated that preoperative RSB was associated with a lower risk of postoperative first time to rescue analgesia (Supplementary Table 1, http://links.lww.com/MD/L974). The number of postoperative times to rescue analgesia did not differ significantly between the groups. However, the Kaplan–Meier curves for the 2 groups were roughly parallel, and a larger sample may have shown significant differences between the 2 groups. Although we had hypothesized that preoperative RSB would reduce the usage of analgesic agents by 20% compared with postoperative RSB, the difference in reduction was approximately 8% in the present study. Based on our results, we recalculated that the number needed to treat in each group was 533 (HR, 0.8; the ratio of not receiving postoperative analgesics, 0.75; 2-sided alpha level, 0.05; power, 0.8).

In addition to its retrospective nature and single-center design, this study had several other limitations. First, we were unable to compare the total postoperative analgesic requirements (frequency of analgesic use and dosage) between the 2 groups. Typically, we compare the total use of a single type of analgesic as a surrogate for postoperative pain assessment.^[3,4,6]^ However, various postoperative analgesics were administered in this study. Therefore, we did not assess the total use of analgesics as comparing findings for different types of analgesics would not have been meaningful. The total frequency of analgesic use and dosage were higher in the pre-RSB group than in the post-RSB group. However, the low risk for first time to rescue analgesia during the initial 24 hours after surgery implied that many patients experienced at least free from pain commonly associated with postoperative discomfort, including nausea, vomiting, sore throat, and fatigue. This result may have contributed to an improvement in the quality of the patient’s recovery. Therefore, our findings, which show that a larger proportion of patients in the pre-RSB group did not require analgesia within 24 hours, can be valuable in determining the appropriate timing for RSB. Second, an accurate time-specific quantitative pain assessment could not be performed. Although a pain score, such as the visual analog scale (VAS), is typically used for this purpose, the VAS was presumed to be unreliable because nurses at our institution did not receive sufficient training to appropriately assess pain. Thus, to avoid incorrect results, we did not assess the VAS scores. Third, the cases in this study involved multi-incision laparoscopic surgeries, and although patients who underwent surgical, urological, and gynecological procedures were selected, the number of surgical incisions and the comparability of the size of surgical trauma in different patients might be among the factors that influenced the study results. Before and after PS matching, the majority of cases (almost 80%) involved fallopian tube or ovarian surgeries, appendectomies, cholecystectomies, and prostatectomies (Supplementary Table 2, http://links.lww.com/MD/L975). In our institution, 4 ports are placed in the umbilical and lower abdomen for fallopian tube and ovarian surgery, 3 in the umbilical and lower abdomen for appendectomies, 4 in the umbilical and upper abdomen for cholecystectomies, and 5 in the periumbilical and lower abdomen for prostatectomies. Therefore, it is expected that in the cases reviewed in this study, the wounds would have been at the umbilical, upper, and lower port sites. This study focused on RSB, which is effective in the umbilical region where pain is likely to be most severe. The technique involves placing a port in the umbilical region. Even with various techniques used, validating the effectiveness of the procedure should not be overly complicated. Fourth, opioids may increase sensitivity to nociceptive stimuli and cause opioid-induced hyperalgesia.^[20]^ High intraoperative doses of remifentanil have been associated with a small but significant increase in acute postoperative pain, and the tendency in this study to administer more remifentanil in the post-RSB group may have influenced the results. Fifth, the anesthesiologists’ experience and skills were unknown. Although RSB was performed under ultrasound guidance, our study was conducted during the transition toward performing RSB with ultrasound, and familiarity with ultrasound-guided RSB was low for some time during the study period. Thus, there may have been some skill-related bias, and future studies should aim to eliminate this bias in their analyses. It is important to note that the specific years of practice of the anesthesiologists who performed the RSB were not recorded, precluding their inclusion in the analysis. Sixth, although oral analgesics may have been prescribed in response to the first time to rescue analgesia, we could not evaluate their use as the records did not include the exact time when the patients received analgesics. However, oral analgesics were rarely prescribed at our institution. Finally, background factors that influence pain remain unknown. Factors, such as anxiety, psychological distress, and preexisting pain, among others, are predictive factors for postoperative pain,^[21]^ but we could not collect the data for these factors. We hope that future prospective studies will be conducted with these data.

## 5. Conclusion

The first time to rescue analgesia tended to be longer and the risk of the first analgesic request occurring within 24 hours was lower in the pre-RSB group than in the post-RSB group. The findings of this study suggest that preoperative RSB may provide preemptive analgesia. Large prospective studies on the timing of RSB has to be conducted to further evaluate these findings.

## Acknowledgments

We would like to thank Editage (www.editage.com) for English language editing.

## Author contributions

**Conceptualization:** Mayuko Nakazawa, Toko Fukushima, Kazuhiro Shoji, Ryo Momosaki.

**Data curation:** Mayuko Nakazawa, Toko Fukushima, Kazuhiro Shoji, Ryo Momosaki.

**Formal analysis:** Mayuko Nakazawa, Toko Fukushima.

**Methodology:** Mayuko Nakazawa, Toko Fukushima.

**Project administration:** Mayuko Nakazawa, Toko Fukushima.

**Visualization:** Mayuko Nakazawa, Toko Fukushima.

**Writing—original draft:** Mayuko Nakazawa, Toko Fukushima, Kazuhiro Shoji, Ryo Momosaki, Yasushi Mio.

**Writing—review & editing:** Mayuko Nakazawa, Toko Fukushima, Kazuhiro Shoji, Ryo Momosaki, Yasushi Mio.

## Supplementary Material






