# Supplementary material for: Preoperative versus postoperative ultrasound-guided rectus sheath block for acute postoperative pain relief after laparoscopy: A retrospective cohort study
Source: Medicine (Baltimore). 2024 Mar 29;103(13):e37597. doi: 10.1097/MD.0000000000037597 (PMC10977526; doi:10.1097/MD.0000000000037597)
Supplement: Supplementary file 1 [file medi-103-e37597-s001.docx]

Supplementary Material(1)

Preoperative versus postoperative ultrasound-guided rectus sheath block for acute postoperative pain relief after laparoscopy: a retrospective cohort study

Mayuko Nakazawa^1,2^, Toko Fukushima^1,2^*, Kazuhiro Shoji^1,2^, Ryo Momosaki^3^, Yasushi Mio^3^

* Correspondence: Toko Fukushima: [j.toko.fukushima105@gmail.co](mailto:j.toko.fukushima105@gmail.co)

**Supplementary Figure 1.** Distribution of propensity scores (PS) in the unmatched (a) and PS-matched groups (b). The PS represents the probability, based on given baseline variables, that any patient in either group would be selected for RSB before and after surgery. Light bars represent the postoperative rectal sheath block group (Post-RSB), and dark bars represent the preoperative rectal sheath block group (Pre-RSB). Overlapping PS scores indicate matched pairs.
