# Supplementary material for: Preoperative versus postoperative ultrasound-guided rectus sheath block for acute postoperative pain relief after laparoscopy: A retrospective cohort study
Source: Medicine (Baltimore). 2024 Mar 29;103(13):e37597. doi: 10.1097/MD.0000000000037597 (PMC10977526; doi:10.1097/MD.0000000000037597)
Supplement: Supplementary file 2 [file medi-103-e37597-s002.docx]

Supplementary Material(2)

Preoperative versus postoperative ultrasound-guided rectus sheath block for acute postoperative pain relief after laparoscopy: a retrospective cohort study

Mayuko Nakazawa^1,2^, Toko Fukushima^1,2^*, Kazuhiro Shoji^1,2^, Ryo Momosaki^3^, Yasushi Mio^3^

* Correspondence: Toko Fukushima: [j.toko.fukushima105@gmail.co](mailto:j.toko.fukushima105@gmail.co)

**Supplementary Table 1.** Patient characteristics and clinical variables before and after propensity-score matching

|  | Before PS matching | | | After PS matching | | |
| --- | --- | --- | --- | --- | --- | --- |
|  | HR | 95% CI | P-value | HR |  | P-value |
| Pre-RSB | 0.82 | [0.63–1.08] | 0.16 | 0.71 | [0.53–0.95] | 0.02 |
| ≥65 y | 0.58 | [0.45–0.74] | 0.00 | 0.59 | [0.41–0.85] | 0.00 |
| Male | 0.73 | [0.56–0.96] | 0.02 | 0.67 | [0.47–0.95] | 0.02 |
| BMI |  |  |  |  |  |  |
| <30 kg/m^2^ | Reference | |  | Reference | |  |
| ≥30 kg/m^2^ | 0.90 | [0.60–1.34] | 0.60 | 0.75 | [0.34–1.69] | 0.49 |
| NA | 1.21 | [0.80–1.81] | 0.37 | 1.17 | [0.76–1.81] | 0.47 |
| Department |  |  |  |  |  |  |
| Gynecology | Reference | |  | Reference | |  |
| General surgery | 0.94 | [0.74–1.19] | 0.59 | 0.85 | [0.63–1.14] | 0.27 |
| Urology | 1.37 | [0.81–2.32] | 0.24 | 1.26 | [0.73–2.17] | 0.40 |
| DAPT | 1.18 | [0.36–3.85] | 0.79 | 1.90 | [1.08–3.34] | 0.03 |
| DOAC | 0.71 | [0.31–1.64] | 0.43 | 0.60 | [0.28–1.31] | 0.20 |
| Emergency | 0.74 | [0.55–0.99] | 0.04 | 0.74 | [0.54–1.01] | 0.06 |
| Fentanyl | 1.61 | [0.60–4.31] | 0.35 | 2.51 | [0.52–12.08] | 0.25 |
| Remifentanil | 1.81 | [1.20–2.73] | 0.00 | 1.80 | [1.20–2.71] | 0.00 |
| Flurbiprofen | 0.95 | [0.39–2.31] | 0.91 | 1.40 | [0.61–3.19] | 0.42 |
| Acetaminophen | 1.33 | [0.50–3.51] | 0.57 | 1.83 | [0.39–8.59] | 0.44 |
| Tramadol | 0.00 | [0.00–Inf] | 0.99 | 0.00 | [0.00–0.00] | 0.00 |
| Duration of anesthesia |  |  |  |  |  |  |
| <120 min | Reference | |  | Reference | |  |
| 120–240 min | 1.37 | [1.11–1.68] | 0.00 | 1.33 | [1.01–1.74] | 0.05 |
| ≥240 min | 1.15 | [0.81–1.62] | 0.44 | 1.06 | [0.65–1.74] | 0.81 |
| Type of anesthesia |  |  |  |  |  |  |
| Sevoflurane | Reference | |  | Reference | |  |
| Desflurane | 0.98 | [0.77–1.24] | 0.85 | 1.08 | [0.78–1.48] | 0.66 |
| TIVA | 0.97 | [0.73–1.28] | 0.80 | 1.01 | [0.67–1.53] | 0.96 |
| Others | 0.49 | [0.12–2.01] | 0.32 | 0.30 | [0.07–1.28] | 0.10 |
| Year |  |  |  |  |  |  |
| 2013 | Reference | |  | Reference | |  |
| 2014 | 0.77 | [0.17–3.57] | 0.74 | 0.65 | [0.16–2.67] | 0.55 |
| 2015 | 0.89 | [0.31–2.55] | 0.83 | 1.10 | [0.57–2.12] | 0.79 |
| 2016 | 0.86 | [0.31–2.40] | 0.78 | 1.33 | [0.69–2.57] | 0.39 |
| 2017 | 0.76 | [0.28–2.10] | 0.60 | 0.93 | [0.53–1.62] | 0.79 |
| 2018 | 0.81 | [0.29–2.25] | 0.68 | 1.07 | [0.58–1.96] | 0.83 |
| 2018 | 0.81 | [0.29–2.25] | 0.68 | 1.07 | [0.58–1.96] | 0.83 |

PS, propensity score; HR, hazard ratio; CI, confidence interval; Pre-RSB, preoperative rectus sheath block; BMI, body mass index; NA, not applicable; DAPT, dual antiplatelet therapy; DOAC, direct oral anticoagulant;; Inf, infinity; ;; TIVA, total intravenous anesthesia
