# Supplementary material for: Preoperative versus postoperative ultrasound-guided rectus sheath block for acute postoperative pain relief after laparoscopy: A retrospective cohort study
Source: Medicine (Baltimore). 2024 Mar 29;103(13):e37597. doi: 10.1097/MD.0000000000037597 (PMC10977526; doi:10.1097/MD.0000000000037597)
Supplement: Supplementary file 3 [file medi-103-e37597-s003.docx]

Supplementary Material(3)

Preoperative versus postoperative ultrasound-guided rectus sheath block for acute postoperative pain relief after laparoscopy: a retrospective cohort study

Mayuko Nakazawa^1,2^, Toko Fukushima^1,2^*, Kazuhiro Shoji^1,2^, Ryo Momosaki^3^, Yasushi Mio^3^

* Correspondence: Toko Fukushima: [j.toko.fukushima105@gmail.co](mailto:j.toko.fukushima105@gmail.co)

**Supplementary Table 2.** Surgical technique before and after propensity-score matching

|  | Before PS matching | | | After PS matching | | |
| --- | --- | --- | --- | --- | --- | --- |
| Factor | Pre-RSB | Post-RSB | SMD | Pre-RSB | Post-RSB | SMD |
| Type of surgery, N |  |  | 0.53 |  |  | 0.23 |
| Fallopian tube or ovary surgery | 85 (37.4) | 95 (24.9) |  | 31 (32.0) | 33 (34.0) |  |
| Appendectomy | 42 (18.5) | 47 (12.3) |  | 18 (18.6) | 13 (13.4) |  |
| Cholecystectomy | 58 (25.6) | 130 (34.0) |  | 28 (28.9) | 25 (25.8) |  |
| Prostatectomy | 0 (0.0) | 31 (8.1) |  | 0 (0.0) | 1 (1.0) |  |
| Others | 42 (18.5) | 79 (20.7) |  | 20 (20.6) | 25 (25.8) |  |

Fallopian tube ovary surgery includes oophorectomy, ovarian cystectomy, salpingectomy, and salpingostomy. PS, propensity score; Pre-RSB, preoperative rectus sheath block; Post-RSB, postoperative rectus sheath block; SMD, standardized mean difference
